# Endoplasmic Reticulum Stress Mediated MDRV p10.8 Protein-Induced Cell Cycle Arrest and Apoptosis Through the PERK/eIF2α Pathway

**DOI:** 10.3389/fmicb.2018.01327

**Published:** 2018-06-21

**Authors:** Quanxi Wang, Xiaoqin Yuan, Yuan Chen, Qingli Zheng, Lihui Xu, Yijian Wu

**Affiliations:** ^1^College of Animal Science, Fujian Agriculture and Forestry University, Fuzhou, China; ^2^Fujian Key Laboratory of Traditional Chinese Veterinary Medicine and Animal Health, Fujian Agriculture and Forestry University, Fuzhou, China

**Keywords:** endoplasmic reticulum stress, MDRV, apoptosis, cell cycle arrest, PERK, eIF2α

## Abstract

In this study, the mechanism of Muscovy duck reovirus (MDRV) p10.8 protein-induced pathogenesis was investigated, with a focus on endoplasmic reticulum (ER) stress. In chicken embryo fibroblasts cell lines (DF1), pCI-neo-flg-p10.8 protein transfection increased the phosphorylation (p-) levels of PERK and eIF2α as shown by Western blotting analysis and led to the dissociation of BiP from PERK as shown by co-immunoprecipitation (Co-IP) analysis. Results of treatment with both ER stress activator and inhibitor further confirmed that p10.8 protein induced ER stress. Subsequently, using flow cytometry analysis, it was also found that p10.8 protein induced cell cycle arrest during the G0/G1 phase. Furthermore, p10.8 transfection increased the phosphorylation levels of PERK and eIF2α, and reduced the expression levels of CDK2, CDK4, and Cyclin E according to Western blotting analysis. Treatment with ER stress activator and ER stress inhibitor after p10.8 protein transfection in DF1 cells further indicated that p10.8 protein induced ER stress, which resulted in cell cycle arrest. The results of knockdown of either PERK or eIF2α genes further confirmed that p10.8 protein-induced ER stress led to cell cycle arrest through the PERK/eIF2α pathway. Further results showed that p10.8 protein induced ER stress and apoptosis in DF1 cells. The expression levels of p-PERK, p-eIF2α, CHOP, cleaved-Caspase12, and cleaved-Caspase3 were increased by p10.8 protein. Test results of treatment with each of Tunicamycin, TUDCA and knockdown of PERK, and eIF2α, confirmed that p10.8 protein induced ER stress involving apoptosis via the PERK/eIF2α pathway. In conclusion, MDRV p10.8 protein induced ER stress that caused cell cycle arrest and apoptosis through the PERK/eIF2α pathway.

## Introduction

Ducklings infected with the reovirus were first reported in 1950 (Kaschula, 1950), and Muscovy duck reovirus (MDRV) was first isolated in 1972 (Gaudry et al., 1972). Furthermore, MDRV infection outbreaks in China have caused serious damage to duckling farms since 1997 (Wu et al., 2001; Wang et al., 2015). Mutation of MDRV is key in the enhancement of its pathogenicity (Cai et al., 2015). The p10.8 gene of MDRV shows huge variation when compared with the Avian reovirus (ARV) p10 gene (Cai et al., 2015). Therefore, it is important to elucidate the biological function of p10.8 in the pathogenesis of MDRV infection.

Polypeptides undergo protein folding in the endoplasmic reticulum (ER). If they are not accurately folded, they are degraded or re-folded in the ER. However, sometimes the unfolded polypeptides accumulate in the ER and cause ER stress (Rashid et al., 2015). To combat the stress, cells then promote the unfolded protein reaction (UPR). UPR is initiated and mediated by three transmembrane stress sensors, protein kinase RNA-like ER kinase (PERK), inositol-requiring protein 1α (IRE1), and activating transcription factor 6 (ATF6) (Schönthal, 2012; Han and Kaufman, 2016; Kroeger et al., 2018). The three sensors are bonded with binding immunoglobulin protein (BiP) during the resting state of cells. Some factors, however, lead to the accumulation of unfolded proteins in ER, such as pathogenic infection, which activates the sensors by dissociating the BiP (Kopp et al., 2018).

The PERK pathway could regulate cellular protein translation. After dissociation with the BiP and PERK forms a homologous dimer and then phosphorylates each other (Hu et al., 2017). Phosphorylated PERK further phosphorylates its downstream protein α-subunit of eukaryotic initiation factor 2 (eIF2α), which is a crucial molecule in the control of protein translation in cells (Liu et al., 2017). So, cellular protein translation usually shuts down when eukaryotic cells are infected by a virus, which suggests the need for the recruitment of ribosomes to translate viral proteins (Hou et al., 2017). This, however, does not improve host cell quality of life, because cells are constantly infected with the virus and their fate is directed toward apoptosis or the cell cycle.

In our previous study, we reported that MDRV activates the death receptor family signaling pathway (Fas, TNFR1), the interleukin receptor signaling pathway (IL1, IL3), the phosphatidylinositol 3-kinase signaling pathway, NF-κB signaling pathway, and the calcium ion signaling pathway to induce apoptosis (Wang et al., 2015). In this study, we aimed to demonstrate and discuss how MDRV p10.8 protein regulates cell survival via the PERK/eIF2α signaling pathway in DF1 cells.

## Materials and Methods

### Virus, Cell Line, and Plasmid Transfection

Muscovy duck reovirus strain YB [MDRV-YB, TCID_50_ = 10^-5.40^, multiplicity of infection (MOI) = 2] was propagated in Muscovy duck embryo fibroblasts (MDEF), which were cultured in RPMI 1640 medium (Hyclone, Logan, UT, United States) supplemented with 2% fetal bovine serum (FBS; Hyclone, Logan, UT, United States), as previously described (Wang et al., 2017a).

The DF1 cell line was grown in Dulbecco’s modified Eagle’s medium (DMEM), supplemented with 10% FBS. Cells, at 70% confluence were transfected with plasmids (pCI-neo-flg, pCI-neo-flg-p10.8) using Lipofectamine 2000 reagent (Promega, Madison, WI, United States), as previously described (Wu et al., 2017a).

### Reagents, Plasmids, and Antibodies

Tunicamycin (TM) and tauroursodeoxycholic acid (TUDCA) were purchased from Sigma (St. Louis, MO, United States). The WesternBright MCF fluorescent Western blotting kit was purchased from Advansta (Menlo Park, CA, United States). Annexin V-FITC was purchased from Beyotime Biotechnology (Shanghai, China). The Co-IP kit was purchased from Promega (Madison, WI, United States). The pCI-neo-flg eukaryotic expression vector was kindly provided by Prof. H. J. Liu (Institute of Molecular Biology, National Chung-Hsing University, Taichung, Taiwan).

BiP Ab, PERK Ab, p-PERK Ab, eIF2α Ab, p- eIF2α Ab, Caspase3 Ab, Cleaved-Caspase3Ab, CHOP Ab, Caspase12 Ab, cleaved-Caspase12 Ab, Cyclin A Ab, Cyclin E Ab, CDK2 Ab, and CDK4 Ab rabbit anti-mouse antibodies were all purchased from Cell Signaling Biotechnology (Beverly, MA, United States). p10.8 Ab was prepared by us through prokaryotic expression and immune rabbit.

### Western Blot

DF1 cell line is the important model cell of avian virus research (Wang et al., 2015; Wu et al., 2017a). DF-1 cells were transfected with plasmids (pCI-neo-flag-p10.8, pCI-neo-flag) or infected with MDRV (MOI = 2) for 24 h, respectively, then were collected and lysed in lysis buffer [50 mM Tris-HCl (pH 7.5), 150 mM NaCl, 1% Nonidet P-40, 0.5% sodium deoxycholate, and 0.1% sodium dodecyl sulfate (SDS), supplemented with a complete protease inhibitor cocktail (Roche, Switzerland)]. A total of 30 μg of protein samples from each treatment were quantified by a Bio-Rad protein assay (Bio-Rad Laboratories, United States), and were separated using 12% SDS-polyacrylamide gel electrophoresis (PAGE) gel. They were then transferred to a PVDF membrane (GE Healthcare Life Sciences). The PVDF membrane was incubated with a primary antibody (BiP, PERK, p-PERK, eIF2α, p-eIF2α, Caspase3, cleaved-Caspase3, CHOP, Caspase12, cleaved-Caspase12, Cyclin A, Cyclin E, CDK2, or CDK4) and the horseradish peroxidase (HRP) conjugated secondary antibody. After incubation with enhanced chemiluminescence (ECL plus) (Amersham/Pharmacia, Buckinghamshire, United Kingdom), the membrane was exposed to X-ray films (Kodak, Rochester, NY, United States). The intensity of target proteins were calculated using Photocapt software (Vilber Lourmat Sté, France). Every protein was tested three times by WB and the intensity of every band was measured three time.

### Co-immunoprecipitation Assays (Co-IP)

In this study, our target was to investigate if p10.8 protein induced BiP dissociation from PERK. Co-immunoprecipitation assays were carried out. Immunoprecipitation was performed using the Catch and Release kit (Upstate Biotechnology) according to the manufacturer’s protocol (Kroeger et al., 2018).

The cells in all groups were scraped off the culture plate, and cracked using a cold cell lysis buffer, then centrifuged at 4°C for 15 min. The supernatant was collected and treated with anti-BiP or anti-PERK antibody, then it was agitated at 4°C for 1 h. The protein A-Sepharose suspension was added and maintained at 4°C for 30 min. Then it was centrifuged at 4°C for 15 min. The protein A-Sepharose mixture was washed five times with NETN (900 mmol/L NaCl). Finally, the samples were analyzed by Western blot (Wu et al., 2017a).

### Treatment of the DF1 Cell Line With an ER Stress Activator and Inhibitor

In our study, the ER stress activator Tunicamycin, and the ER stress inhibitor Tauroursodeoxycholic acid, were applied to control the level of ER stress in cells. DF1 cells were subcultured in 6-well or 12-well plates for 12 h. In the next step, we either left the medium unchanged, incubated it with MDRV YB strain for 0.5 h, or transfected it with pCI-neo-flg-p10.8 or pCI-neo-flg, and then added complete DMEM medium containing 10% serum and Tunicamycin (TM, 2 μg/mL) or Tauroursodeoxycholic acid (TUDCA,2 μg/mL) for 24 h. The protein expression of p10.8, BiP, PERK, p-PERK, eIF2α, p-eIF2α, Caspase3, Caspase12, cleaved-Caspase12, cleaved-Caspase3, and CHOP were all detected by Western blot. The percentage of apoptotic cells and cells with cell cycle arrest were detected by flow cytometry.

### Cell Cycle and Apoptosis Detected by Flow Cytometry

After a 24 h transfection, the medium was removed and cells were washed twice with PBS (pH 7.0). DF1 cells were digested using 0.25% pancreatin for 3–5 min. Then they were re-suspended and were transferred into a centrifuge tube. Cells were centrifuged at 1000 *g* for 5 min, and were then fixed with 70% cold ethanol at 4°C for 2 h. Subsequently, they were centrifuged again and washed thrice in PBS. Finally, cells were stained with PI or LAnnexin V-FITC dye containing a final concentration of 100 g/mLRNaseA at 37°C for 30 min. Cell cycle or apoptosis were analyzed by flow cytometry (BD Calibur) (Wang et al., 2017a).

### Gene Silencing

Specific siRNA oligonucleotides of PERK and eIF2α were synthesized by Biomics (Biomics Biotechnology, Co., Ltd., Nantong, China), respectively. The sequences of oligonucleotides were as follows:

siPERK-1-F: 5′-GCGAGGAUGUUGUCUUAGUdTdT-3′,
siPERK-1-R: 5′-ACUAAGACAACAUCCUCGCdTdT-3′,
siPERK-2-F: 5′-CCAGUGUCUAUUUGGGUAUdTdT-3′,
siPERK-2-R: 5′-AUACCCAAAUAGACACUGGdTdT-3′,
siPERK-3-F: 5′-CAACCUUUAUUGUACGCAAdTdT-3′,
siPERK-3-R: 5′-UUGCGUACAAUAAAGGUUGdTdT-3′,
sieIF2α-1-F: 5′-GUCCAGAAGACGUAUUCGUdTdT-3′,
sieIF2α-1-R: 5′-ACGAAUACGUCUUCUGGACdTdT-3′,
sieIF2α-2-F: 5′-GGUUGCGUGUUAUGGUUAUdTdT-3′,
sieIF2α-2-R: 5′-AUAACCAUAACACGCAACCdTdT-3′,
sieIF2α-3-F: 5′-GCCUGGGUAUUUGAUGACAdTdT-3′,
sieIF2α-3-R: 5′-UGUCAUCAAAUACCCAGGCdTdT-3′.

DF1 cells were prepared in 6-well plates. These specific siRNA oligonucleotides were transfected into DF1 cells using Lip2000. The protein expression of PERK and eIF2α were determined by Western blot. The optional PERK- or eIF2α-specific siRNA oligonucleotides (siPERK-1, sieIF2α-1; **Supplementary Figure S1**) were used to evaluate the effects of p10.8-induced DF1 cell apoptosis and cell cycle arrest.

Five groups of DF1 cells were prepared in 6-well plates. The first group was mock (control); the second one was transfected with pCI-neo-flag; the third was transfected with pCI-neo-flag-p10.8; the fourth was transfected with siPERK-1 (or sieIF2α-1) and after 6 h transfected with pCI-neo-flag-p10.8; the fifth was transfected with siPERK-1 (or sieIF2α-1). At 24 h post-transfection, cells were collected and total proteins were extracted. The protein expression levels of p10.8, BiP, PERK, p-PERK, eIF2α, p-eIF2α, Caspase3, Caspase12, cleaved-Caspase12, cleaved-Caspase3, and CHOP were analyzed by Western blot.

### Statistical Analysis

Statistic Package for Social Sciences (SPSS) 13.0 for Windows (SPSS, Inc., Chicago, IL, United States) was used to analyze data. The results were expressed as mean ± SEM. Statistical analyses were performed using the non-parametric Comparisons Test and Student’s *t*-test. When the *P*-value was < 0.05, the difference was regarded as statistically significant. When the *P*-value was < 0.01, the difference was extremely significant.

## Results

### MDRV p10.8 Protein Induced ER Stress in DF1 Cells

**FIGURE 1:**
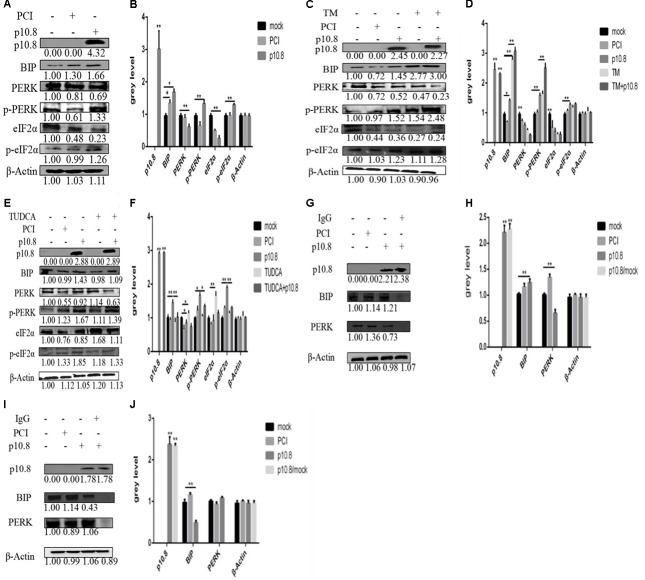
Muscovy duck reovirus (MDRV) p10.8 protein induced ER stress in DF1 cells. In order to investigate whether MDRV p10.8 protein induced endoplasmic reticulum (ER) stress in DF1 cells **(A,B)** at 24 h post-transfection, Western blot was performed to determine the protein (p10.8, BiP, PERK, p-PERK and eIF2α, p-eIF2α) expression in the three groups of DF1 cells (mock, pCI, and p10.8), and β-Actin was used as the reference gene (the same as in the following study). The gray value of all bands was analyzed by Image J in SPSS (^∗^*P* < 0.05, ^∗∗^*P* < 0.01, the same as in the following study). **(C,D)** DF1 cells were treated with or without Tunicamycin (TM; final concentration 1 mmol/L) after transfection with pCI-neo-flg-p10.8, mock and eukaryotic expression plasmid transfection (pCI) as the control. At 24 h post-transfection, Western blot was used to determine the protein (p10.8, BiP, PERK, p-PERK, eIF2α, or p-eIF2α) expression in the five groups; gray values were measured and analyzed. **(E,F)** Cells were also treated with or without TUDCA (final concentration 1 mmol/L) after transfection with pCI-neo-flg-p10.8; protein expression was analyzed by Western blot, and the expression levels were analyzed. **(G–J)** In order to investigate whether the p10.8 protein could regulate the disaggregation of the complex substance BiP-PERK and Co-IP were used to arrest BiP-PERK. Western blot was used to detect the complex substance with anti-BiP antibody, anti-PERK antibody, and BiP, PERK negative antibody, respectively, at 24 h post-transfection in DF1 cells; gray values were measured and analyzed.

When DF1 cells were transfected with pCI-neo-flg-p10.8 for 24 h, the Western blot analyses showed that the expression levels of BiP, p-PERK, and p-eIF2α were significantly increased and PERK, eIF2α were significantly decreased, compared with the control (**Figures 1A,B**). When synergistic treatment of p10.8 protein and TM on DF1 cells was performed, the expression levels of BiP, p-PERK, and p-eIF2α were significantly increased, when compared to the treatments with either p10.8 or TM only (**Figures 1C,D**). Furthermore, when DF1 cells were treated with combined p10.8 protein and TUDCA, the expression levels of BiP, p-PERK, and p-eIF2α were significantly reduced, as compared to the treatments with either p10.8 only or TM only (**Figures 1E,F**). Protein Co-IP analysis showed that p10.8 protein caused BiP to dissociate from PERK and increased the phosphorylation levels of PERK and eIF2α, which led to the progression of ER stress in DF1 cells (**Figures 1G–J**). These results indicated that MDRV p10.8 protein could induce ER stress in the DF1 cell line.

### MDRV p10.8 Protein Induced Cell Cycle Arrest via the BiP/PERK/eIF2α/CDKs Pathway

**FIGURE 2:**
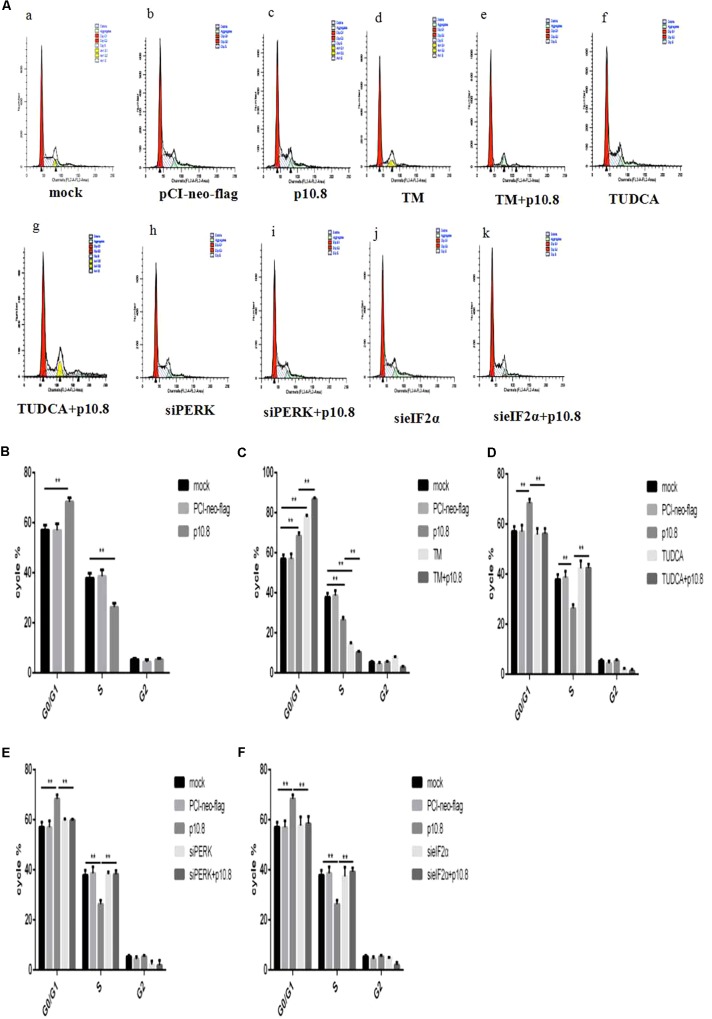
Cell cycle detected by flow cytometry. MDRV p10.8 protein-induced cell cycle arrest was investigated in DF1 cells. Cells were treated with (a) mock, (b) plasmid pCI-neo-flg, (c) recombinant plasmid pCI-neo-flg-p10.8, (d) 2 μg/mL TM, (e) pCI-neo-flg-p10.8+TM, (f) 2 μg/mL TUDCA, (g) pCI-neo-flg-p10.8+TUDCA, (h) siPERK, (i) pCI-neo-flg-p10.8+siPERK, (j) sieIF2α, and (k) pCI-neo-flg-p10.8+sieIF2α, respectively. After 24 h, the proportion of cells at three phases (G1, S, and G2) were detected by flow cytometry. **(A)** Images of cell cycle arrest in DF1 cells as determined by flow cytometry. Statistical analysis relating to the proportion of cells at three phases treated with **(B)** p10.8, **(C)** TM and pCI-neo-flg-p10.8+TM, **(D)** TUDCA and pCI-neo-flg-p10.8+TUDCA, **(E)** siPERK and pCI-neo-flg-p10.8+siPERK, **(F)** sieIF2α and pCI-neo-flg-p10.8+sieIF2α compared with mock and pCI-neo-flg (G1, S, and G2). ^∗^*P* < 0.05, ^∗∗^*P* < 0.01, the same as in the following study.

**FIGURE 3:**
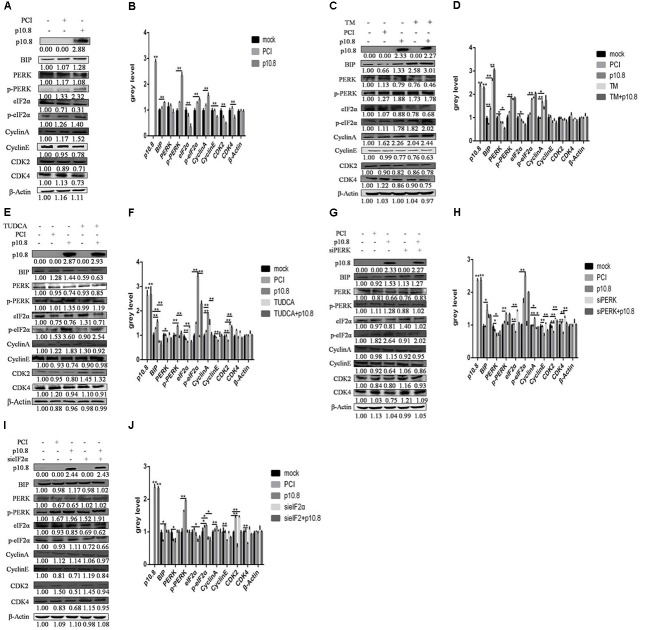
Muscovy duck reovirus p10.8 protein induced cell cycle arrest via the BiP/PERK/eIF2α/CDK pathway. **(A,B)** Protein p10.8 expression, ER index protein (BiP, PERK, p-PERK, eIF2α, and p-eIF2α) expression and cell cycle regulational protein (Cyclin A, Cyclin E, CDK2, and CDK4) expression in three groups of DF1 cells (mock, pCI, and p10.8) were analyzed by Western blot; β-Actin was the reference gene (the same as in the following study). Expression levels were statistically analyzed (^∗^*P* < 0.05, ^∗∗^*P* < 0.01, the same as in the following study). **(C,D)** Cells were treated with or without Tunicamycin (TM, final concentration 1 mmol/L) after transfection with pCI-neo-flg-p10.8, mock and eukaryotic expression plasmid transfection (pCI) as the control. At 24 h post-transfection, the proteins of p10.8, ER index proteins, and cell cycle regulational proteins were determined by Western blot in the five groups, and gray values were measured and statistically analyzed. **(E,F)** More cells were also treated with or without TUDCA (final concentration 1 mmol/L) after transfection with pCI-neo-flg-p10.8, the protein (p10.8, BiP, PERK, p-PERK, eIF2α, p-eIF2α, Cyclin A, Cyclin E, CDK2, and CDK4) expression was analyzed by Western blot, and the expression levels were analyzed. **(G–J)** siPERK and sieIF2α were used to knockdown PERK and eIF2α expression; images of protein expression are shown, and gray values were measured and statistically analyzed.

In the first experiment, we aimed to confirm whether p10.8 induced cell cycle arrest. After the transfection of pCI-neo-flg-p10.8 for 24 h in the DF1 cell line, cell cycle phases were detected by flow cytometry. The results showed that after a 24 h transfection, p10.8 protein had significantly increased the proportion of G0/G1 phase cells (**Figures 2A,B**). It was therefore considered that p10.8 protein could induce cell cycle arrest at the G0/G1 phase. Then the expression levels of BiP, PERK, p-PERK, eIF2α and p-eIF2α, CyclinE, CDK2, and CDK4 in DF1 cells were analyzed by Western blot. Results showed that at 24 h after transfection with p10.8 protein, the expression levels of BiP, PERK, p-PERK, eIF2α, and p-eIF2α were significantly increased, and the expression levels of Cyclin E, CDK2, and CDK4 were significantly reduced as compared with cells in the control group (**Figures 3A,B**). These results indicated that MDRV p10.8 protein could induce cell cycle arrest at the G0/G1 phase in DF1 cells.

In order to elucidate whether the p10.8 protein induced ER stress and caused cell cycle arrest, it was co-treated with TM or TUDCA in DF1 cells. The co-treatment with TM significantly increased the proportion of cells in the G0/G1 phase (**Figures 2A,C**) and significantly reduced the expression of Cyclin E, CDK2, and CDK4 (**Figures 3C,D**). Furthermore, the co-treatment with TUDCA significantly reduced the proportion of G0/G1 phase cells (**Figures 2A,D**) and significantly increased the expression of Cyclin E, CDK2, and CDK4 (**Figures 3E,F**), as compared with the p10.8 protein only treatment. Therefore, we considered that p10.8 protein induced cell cycle arrest through ER stress.

In order to further prove that p10.8 protein induced cell cycle arrest through the PERK/eIF2α pathway, the effects of these genes on RNA was investigated. Results showed that after knockdown of the PERK or eIF2α gene in DF1 cells and co-transfection with p10.8 protein, the proportion of G0/G1 phase cells was significantly reduced (**Figures 2A,E,F**) and the expression of Cyclin E, CDK2, and CDK4 protein were all significantly increased (**Figures 3G–J**), as compared to transfection with p10.8 protein only. These results suggested that p10.8 protein induced cell cycle arrest via the PERK/eIF2α pathway of ER stress.

### MDRV p10.8 Protein Induced Apoptosis via the BiP/PERK/eIF2α/CHOP Pathway

Our previous study demonstrated that the induction of apoptosis is a crucial pathogenic mechanism of MDRV infection (Wang et al., 2017a). In this paper, our target was to investigate how ER stress-mediated apoptosis induced by the MDRV p10.8 protein.

**FIGURE 4:**
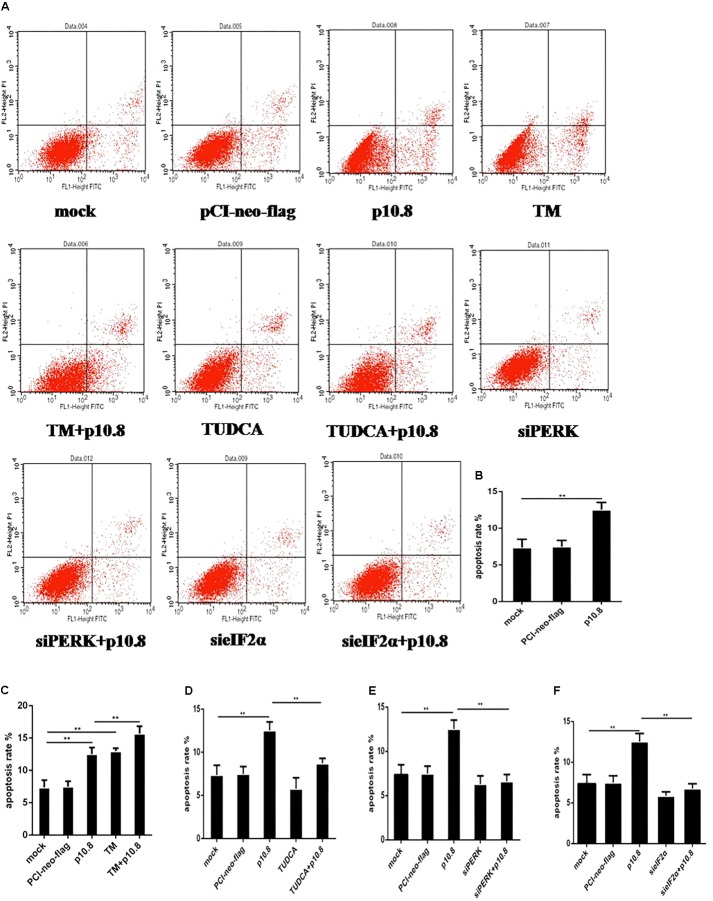
Apoptosis was detected by flow cytometry. MDRV p10.8 protein-induced cell apoptosis was investigated in DF1 cells treated with (a) mock, (b) plasmid pCI-neo-flg, (c) recombinant plasmid pCI-neo-flg-p10.8, (d) 2 μg/mL TM, (e) pCI-neo-flg-p10.8+TM, (f) 2 μg/mL TUDCA, (g) pCI-neo-flg-p10.8+TUDCA, (h) siPERK, (i) pCI-neo-flg-p10.8+siPERK, (j) sieIF2α, (k) pCI-neo-flg-p10.8+sieIF2α, respectively. After 24 h, cells were stained with Annexin V-FITC/PI and the proportion of apoptotic cells in each group were detected by flow cytometry. **(A)** Images of apoptosis were determined via flow cytometry in DF1 cells. Statistical analysis of the proportions of cell apoptosis in **(B)** p10.8, **(C)** TM and pCI-neo-flg-p10.8+TM, **(D)** TUDCA and pCI-neo-flg-p10.8+TUDCA, **(E)** siPERK and pCI-neo-flg-p10.8+siPERK, **(F)** sieIF2α and pCI-neo-flg-p10.8+sieIF2α compared with mock and pCI-neo-flg. ^∗^*P* < 0.05, ^∗∗^*P* < 0.01, the same as in the following study.

**FIGURE 5:**
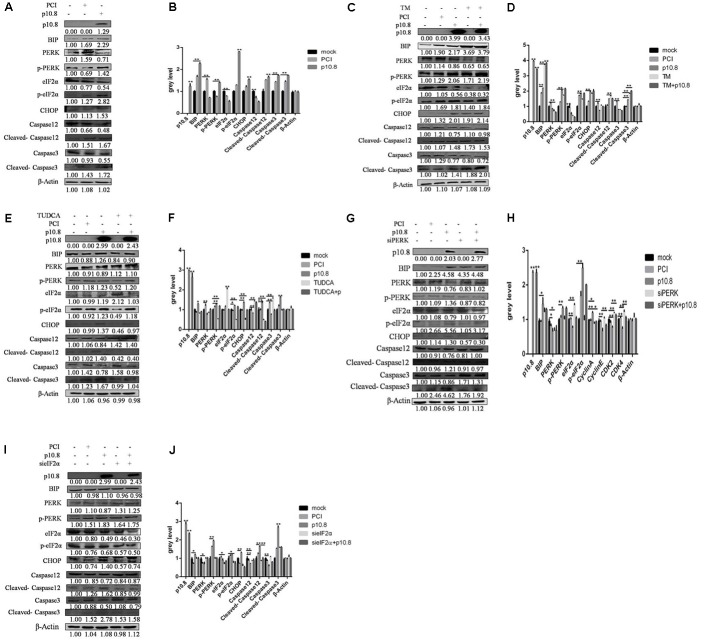
Muscovy duck reovirus p10.8 protein induced apoptosis via the BiP/PERK/eIF2α/CHOP pathway. **(A,B)** Protein p10.8 expression, ER index protein (BiP, PERK, p-PERK, eIF2α, and p-eIF2α) expression, and apoptosis index protein (CHOP, Caspase12, cleaved-Caspase12, Caspase3, and cleaved-Caspase3) expression in three groups of DF1 cells (mock, pCI, and p10.8) were analyzed by Western blot; β-Actin was used as the reference gene (the same as in the following study). Expression levels were statistically analyzed (^∗^*P* < 0.05, ^∗∗^*P* < 0.01, the same as in the following study). **(C,D)** Cells were treated with or without Tunicamycin (TM, final concentration 1 mmol/L) after transfection with pCI-neo-flg-p10.8, mock, and eukaryotic expression plasmid transfection (pCI) as the control. At 24 h post-transfection, the proteins of p10.8, ER index proteins, and apoptosis index proteins were determined by Western blot in the five groups, and the gray values were measured and statistically analyzed. **(E,F)** Further cells were treated with or without TUDCA (final concentration 1 mmol/L) after transfection with pCI-neo-flg-p10.8, protein (p10.8, BiP, PERK, p-PERK, eIF2α, p-eIF2α, CHOP, Caspase12, cleaved-Caspase12, Caspase3, and cleaved-Caspase3) expression was analyzed by Western blot, and expression levels were analyzed. **(G–J)** siPERK and sieIF2α were used to knockdown PERK and eIF2α expression, the images of protein expression were represented, and the gray values were measured and statistically analyzed.

Firstly, it was found that the percentage of apoptotic cells in the DF1 cell line was significantly increased by flow cytometry analysis, at the post-transfection of pCI-neo-flg-p10.8, in comparison with post-transfection control vector and mock cells (**Figures 4A,B**). Furthermore, Western blot analysis showed that p10.8 protein could increase the protein expression of BiP, p-PERK, p-eIF2α, CHOP, cleaved-Caspase12, and cleaved-Caspase3 in DF1 cells (**Figures 5A,B**), indicating that p10.8 protein induced cell apoptosis related to ER stress.

Subsequently, the p10.8 protein was co-treated with TM or TUDCA in DF1 cells. Results showed that the percentage of apoptotic cells in the DF1 cell line co-treated with p10.8 and TM was significantly increased, compared with the p10.8 protein transfection only (**Figures 4A,C**). Moreover, the percentage of apoptotic cells in the DF1 cell line co-treated with p10.8 and TUDCA was significantly reduced, compared with the p10.8 protein transfection only (**Figures 4A,D**). Western blot analysis showed that the protein expression of BiP, p-PERK, p-eIF2α, CHOP, cleaved-Caspase12, and cleaved-Caspase3 were increased in DF1 cells co-treated with p10.8 and TM, as compared with p10.8 protein transfection only (**Figures 5C,D**). Furthermore, the protein expression of BiP, p-PERK, p-eIF2α, CHOP, cleaved-Caspase12, and cleaved-Caspase3 were reduced in DF1 cells co-treated with p10.8 and TUDCA, compared with the sole p10.8 protein transfection (**Figures 5E,F**). These results further confirmed that p10.8 protein induced apoptosis via ER stress.

In order to further prove whether p10.8 induced apoptosis took place via the PERK/eIF2α pathway, RNA interference in the PERK and eIF2α genes was carried out in this study. It was found that following knockdown of either the PERK or eIF2α genes by transfection of pCI-neo-flg-p10.8, the percentage of apoptotic cells in the DF1 cell line co-treated with p10.8 and TUDCA was significantly reduced, compared with the p10.8 protein transfecti on only (**Figures 4E,F**), and the protein expression of CHOP, cleaved-Caspase12, and cleaved-Caspase3 were significantly decreased (**Figures 5G–J**); this indicated that p10.8 protein induced apoptosis occurred through the PERK/eIF2α pathway.

### MDRV-Induced ER Stress to Apoptosis and Cell Cycle Arrest Through the PERK/eIF2 Pathway

**FIGURE 6:**
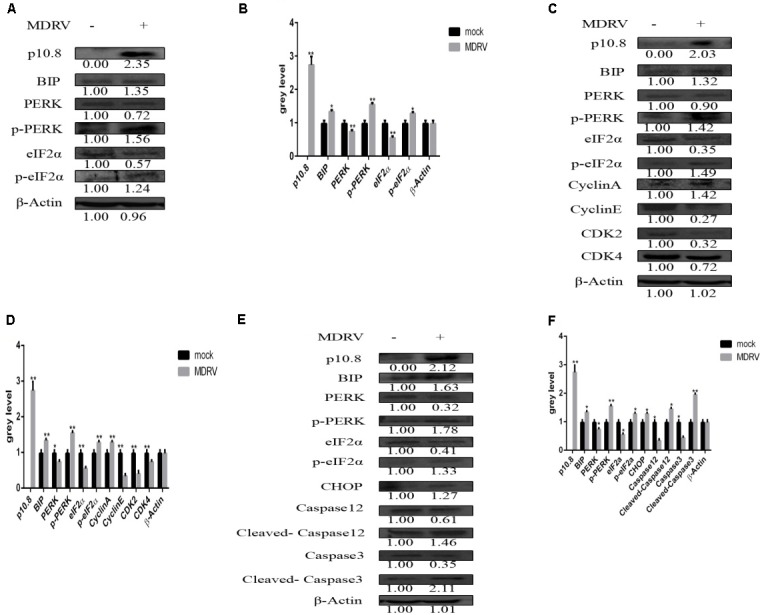
Muscovy duck reovirus induced cell cycle arrest and apoptosis through ER stress. **(A,B)** Two groups of DF1 cells were subcultured in 6-well plates for 24 h. The first group was mock, the second was transfected with MDRV. At 24 h post-transfection, protein (p10.8, BiP, PERK, p-PERK, eIF2α, and p-eIF2α) expression was analyzed by Western blot; β-Actin was used as the reference gene (the same as in the following study). The expression levels were statistically analyzed (^∗^*P* < 0.05, ^∗∗^*P* < 0.01, the same as in the following study). **(C,D)** Protein p10.8, BiP, PERK, p-PERK, eIF2α, p-eIF2α, Cyclin A, Cyclin E, CDK2, and CDK4 expression was analyzed by Western blot, and the expression levels were statistically analyzed. **(E,F)** Protein p10.8, BiP, PERK, p-PERK, eIF2α, p-eIF2α, CHOP, Caspase12, cleaved-Caspase12, Caspase3, and cleaved-Caspase3 expression was analyzed by Western blot; β-Actin was used as the reference gene, and the expression levels were statistically analyzed.

Finally, MDRV whole virus induced ER stress involving apoptosis and cell cycle arrest were investigated in the DF1 cell line. The results showed that MDRV infection significantly increased the protein expression of BiP, p-PERK, and p-eIF2α (**Figures 6A,B**). In addition, the protein expression of Cyclin E, CDK2, and CDK4 were significantly reduced in MDRV infected DF1 cells, compared with mock cells (**Figures 6C,D**). Furthermore, the expression of apoptosis related protein CHOP, cleaved-Caspase12, and cleaved-Caspase3 were significantly increased in MDRV infected DF1 cells, compared with mock cells (**Figures 6E,F**). The results indicated that MDRV infection induced apoptosis and cell cycle arrest were associated with the PERK/eIF2 α pathway.

## Discussion

Recently, great progress has been made into the pathogenic mechanisms of MDRV infection. Studies examining the spleen transcriptome profile of Muscovy ducklings in response to MDRV indicate that this reovirus could induce innate immune function through four signal pathways: the Janus kinase-signal transducer, activator of transcription signaling pathway (JAK-STAT), the retinoic acid-inducible gene I (RIG-I)-like, and Toll-like receptor (TLR) signaling pathways (Wu et al., 2017b). Wang reports that MDRV infection inhibits cholesterol efflux from hepatic cells and reduces the expression of key enzymes involved in fatty acid degradation (scavenger receptor class b type 1, ABCG8, and APOA4), leading to the accumulation of fatty acids and cholesterol in liver cells (Wang et al., 2017b). The molecular mechanism of apoptosis induced by MDRV has been revealed by transcriptomic analysis, which shows that MDRV could activate the fatty acid synthase signaling pathway, the interleukin 1 receptor signaling pathway, and the phosphatidylinositol 3-kinase signaling pathway to induce apoptosis (Wang et al., 2017a). MDRV could cause injury of the small intestinal mucosal immune barrier and mucosal immune function in sick Muscovy ducklings (Wu et al., 2017b). MDRV σNS protein could trigger autophagy to enhance its replication (Wu et al., 2017a). The current study revealed that MDRV could induce ER stress and that p10.8 protein was the critical factor; these results provided new evidence for elucidating the molecular pathogenic mechanism of MDRV.

Many viruses are known to induce cell apoptosis and cell cycle arrest, and are also associated with ER stress, which provides an appropriate intracellular environment for its replication (Lin et al., 2015; Zhou Y. et al., 2017). Viruses that utilize host cell ER as an integral part of their life cycle are predicted to cause some level of ER stress (Khongwichit et al., 2016). Furthermore, virus infection is related to UPR and also to apoptosis and cell cycle arrest (Jheng et al., 2014). Bovine viral diarrhea virus (BVDV) and the related flaviviruses use host ER as the primary site of envelope glycoprotein biogenesis, genomic replication, and particle assembly (Jordan et al., 2002). Porcine circovirus 2 (PCV2) deploys UPR to enhance its replication (Zhou et al., 2016). Classic swine fever virus NS2 protein induces ER stress, modulates cellular growth and cell cycle progression through the induction of S-phase arrest and provides a cellular environment that is beneficial for viral replication (Tang et al., 2010). Porcine epidemic diarrhea virus (PEDV) N protein localizes in the ER, inhibits intra-epithelial carcinoma cell growth and prolongs the S-phase of the cell cycle (Xu et al., 2013). The current study revealed that MDRV p10.8 protein induced ER stress and modulated DF1 cell line apoptosis and cell cycle arrest at the G0/G1-phase by addition ER stress agonist and inhibitor with p10.8 protein transfection, which provided novel information on the function of MDRV p10.8 protein.

Many viruses mediate host cell apoptosis and cell cycle arrest through the PERK/eIF2α pathway, an important branch of ER stress. PERK detects unfolded proteins in the ER. Activation of PERK leads to phosphorylation of the alpha-subunit of eIF2α, which inhibits the exchange of eIF2-GDP for eIF2-GTP (Zhou et al., 2016). The phosphorylation of eIF2α decreases the level of active eIF2/tRNAiMet/GTP ternary complex, leading to the reversible inhibition of translation initiation (Kim et al., 2017). In the present study, DF1 cells were transfected with p10.8 protein after treatment with TM or TUDCA; the results of the phosphorylation of PERK and eIF2α suggested that MDRV p10.8 protein induced ER stress in DF1 cells.

The PERK-eIF2α-CDK branch of the UPR pathway plays a role in virus-induced cell cycle arrest. After UPR activation, cell-cycle arrest occurs primarily in the G0/G1 phase (Zhou Y.S. et al., 2017). Progression through the G0/G1 phase requires the activity of cyclin D in association with either CDK4 or CDK6, followed by activation of the cyclin E- and A-dependent kinase CDK2, as cells approach the G1/S transition (Sherr, 1994; Joseph et al., 2000). In our study, Co-IP results showed p10.8 transfection caused the dissociation of Bip from PERK and activated PERK. In addition, results of the expression inhibition of PERK and eIF2α revealed that p10.8 protein caused cell cycle arrest at the G0/G1 phase via the PERK-eIF2α-CDK pathway.

The branch of the UPR pathway plays a role in virus-induced apoptosis. C/EBP homologous protein (CHOP), a proapoptotic transcription factor, is activated by ER stress (Tao et al., 2016). The activation of PERK and eIF2α are required for CHOP induction (Gu et al., 2017). Persistent PCV2 infection could lead to selective activation of PERK via the PERK-eIF2α-ATF4-CHOP axis (Zhou et al., 2016); PCV2 Cap protein induces UPR and apoptosis via the PERK/eIF2α/ATF4/CHOP/Bcl-2 pathway (Zhou Y.S. et al., 2017). Furthermore, the hepatitis B virus X protein (HBx) suppresses eIF2α phosphorylation, inhibiting expression of ATF4/CHOP/Bcl-2 (Li et al., 2017). In the present study, knockdown of PERK or eIF2α ameliorated CHOP expression induced by MDRV p10.8 protein, suggesting that the activation of PERK and eIF2α are required for CHOP induction. Previously, MDRV p10.8 protein has been shown to be involved in cell apoptosis (Guo et al., 2014). However, the means by which p10.8 protein induces apoptosis is little understood. In the current study, p10.8 protein was found to induce DF1 cells toward apoptosis via the PERK-eIF2α-CHOP pathway. TM or TUDCA, si-PERK, and si-eIF2α were used to support the above mentioned results.

Our study revealed a regulatory network between MDRV p10.8 protein and ER stress-mediated cell cycle arrest and apoptosis. The p10.8 protein is a non-structural protein of MDRV and is coded for by the s4 gene. There is no membrane-spanning domain and function of fusion protein in the p10.8 protein, thus differing from the ARV p10 protein. It is therefore very important to elucidate the biological function of p10.8 in the pathogenesis of MDRV infection. The MDRV p10.8 protein induced significant ER stress in DF1 cells, which led to the activation of PERK and then to phosphorylation of the alpha-subunit of eIF2α. The p-eIF2α subsequently inhibited translation of cell cycle mediation proteins such as CDK2 and CDK4, and then activated apoptotic mediation proteins such as CHOP or Caspase3. At present, there are no cell lines available from duck tissues. DF1 is an important cell line for research relating to avian viruses. Consequently, functional research relating to duck viruses are carried out in the DF1 cell line (Guo et al., 2014; Wu et al., 2017a).

In the current study, we investigated cell cycle arrest and apoptosis induced by the p10.8 protein via ER stress. We also investigated cell cycle arrest and apoptosis induced by MDRV. Following 24 h of MDRV infection, protein expression levels of p10.8, BiP, p-PERK, p-eIF2α, CHOP, cleaved-Caspase12, cleaved-Caspase3 all increased, while expression levels of Cyclin E, CDK2, CDK4 decreased. These results indicate that MDRV can induce cell cycle arrest and apoptosis, and were associated with ER stress. Therefore, our results demonstrate that the effects elicited by p10.8 protein transfection (ER stress, cell cycle arrest, and apoptosis) also occurred following infection with MDRV.

## Author Contributions

QW, XY, and YC performed most of the experiments. QZ, LX, and YW analyzed the data. QW conceived and designed the experiments, wrote the paper, and supervised the project. All authors reviewed the manuscript.

## Conflict of Interest Statement

The authors declare that the research was conducted in the absence of any commercial or financial relationships that could be construed as a potential conflict of interest.

## Supplementary Material

FIGURE S1 Interference effect of specific siRNA oligonucleotides in DF1 cells. siRNA oligonucleotides (siPERK-1, siPERK-2 and siPERK-3, sieIF2α-1, sieIF2α-2, and sieIF2α-3) were transfected into DF1 cells, respectively. **(A,C)** At 24 h post-transfection, protein PERK or eIF2α expression was analyzed by Western blot; β-Actin was used as the reference gene. **(B,D)** Expression levels were statistically analyzed. ^∗^*P* < 0.05, ^∗∗^*P* < 0.01, the same as in the following study.
