# Supplementary figures and images for: Endoplasmic Reticulum Stress Mediated MDRV p10.8 Protein-Induced Cell Cycle Arrest and Apoptosis Through the PERK/eIF2α Pathway
Source: Front Microbiol. 2018 Jun 21;9:1327. doi: 10.3389/fmicb.2018.01327 (PMC6021497; doi:10.3389/fmicb.2018.01327)

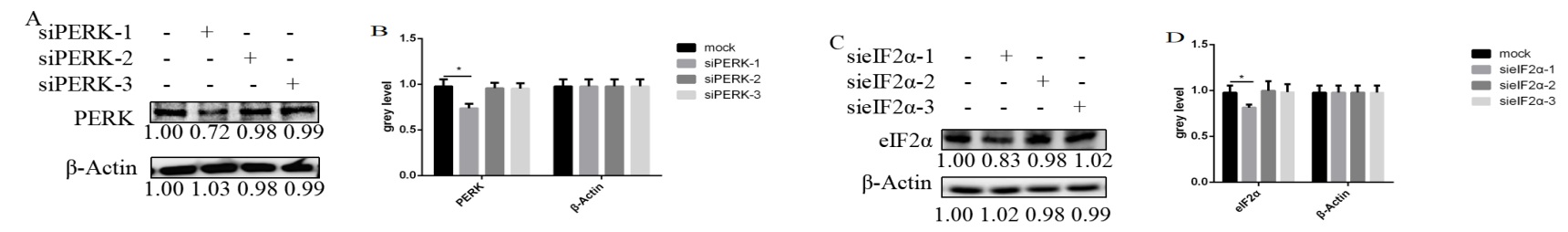

Supplement: FIGURE S1 — Interference effect of specific siRNA oligonucleotides in DF1 cells. siRNA oligonucleotides (siPERK-1, siPERK-2 and siPERK-3, sieIF2α-1, sieIF2α-2, and sieIF2α-3) were transfected into DF1 cells, respectively. (A,C) At 24 h post-transfection, protein PERK or eIF2α expression was analyzed by Western blot; β-Actin was used as the reference gene. (B,D) Expression levels were statistically analyzed. ∗P < 0.05, ∗∗P < 0.01, the same as in the following study. [file Image_1.JPEG]
